# Computational modeling of the EGFR network elucidates control mechanisms regulating signal dynamics

**DOI:** 10.1186/1752-0509-3-118

**Published:** 2009-12-22

**Authors:** Dennis YQ Wang, Luca Cardelli, Andrew Phillips, Nir Piterman, Jasmin Fisher

**Affiliations:** 1 MRC Biostatistics Unit, University of Cambridge, Cambridge, UK; 2 Microsoft Research, Cambridge, UK; 3 Department of Computing, Imperial College London, UK

## Abstract

**Background:**

The epidermal growth factor receptor (EGFR) signaling pathway plays a key role in regulation of cellular growth and development. While highly studied, it is still not fully understood how the signal is orchestrated. One of the reasons for the complexity of this pathway is the extensive network of inter-connected components involved in the signaling. In the aim of identifying critical mechanisms controlling signal transduction we have performed extensive analysis of an executable model of the EGFR pathway using the stochastic pi-calculus as a modeling language.

**Results:**

Our analysis, done through simulation of various perturbations, suggests that the EGFR pathway contains regions of functional redundancy in the upstream parts; in the event of low EGF stimulus or partial system failure, this redundancy helps to maintain functional robustness. Downstream parts, like the parts controlling Ras and ERK, have fewer redundancies, and more than 50% inhibition of specific reactions in those parts greatly attenuates signal response. In addition, we suggest an abstract model that captures the main control mechanisms in the pathway. Simulation of this abstract model suggests that without redundancies in the upstream modules, signal transduction through the entire pathway could be attenuated. In terms of specific control mechanisms, we have identified positive feedback loops whose role is to prolong the active state of key components (e.g., MEK-PP, Ras-GTP), and negative feedback loops that help promote signal adaptation and stabilization.

**Conclusions:**

The insights gained from simulating this executable model facilitate the formulation of specific hypotheses regarding the control mechanisms of the EGFR signaling, and further substantiate the benefit to construct abstract executable models of large complex biological networks.

## Background

Important cellular processes, such as differentiation and division, rely on signal transduction cascades that occur throughout the cell. The signal transduction cascade, initiated by the binding of epidermal growth factor (EGF) to the epidermal growth factor receptor (EGFR), is essential for normal cell growth and development [1]. Conversely, aberrant activity of the EGFR signaling cascade has been shown to play a key role in tumor cell growth and cell differentiation [2]. Due to the complex inter-connectivity of the EGFR signaling network, it is difficult to identify conserved signaling modules and those specific control mechanisms that modulate the strength of EGFR signaling [3]. The high coupling of intermediate components within this signaling network has limited our ability to understand their functionality and how they affect the robustness of the overall pathway [4].

The EGFR signaling cascade is initiated by the binding of EGF to the transmembrane EGFR at its extracellular domain. The binding induces dimerization and autophosphorylation of its intracellular domains. The dimers form docking sites for the recruitment of cytoplasmic enzymes and adapter proteins, starting a complex signaling cascade [5]. The subsequent internalization and dissociation of the signaling complexes lead to activation of Ras-GTP by hydrolization of Ras-GDP [6]. There are two principle pathways leading to this activation, the Shc-dependent and Shc-independent pathways. The switch-like Ras-GTP plays a significant role in stimulating the mitogen activated protein kinase (MAPK) signaling cascade [7]. The end result of the cascade is phosphorylated ERK proteins that go on to regulate several cellular proteins and nuclear transcription factors important for cell growth and differentiation [4]. In this way, the EGFR signaling cascade can be explained as being composed of various well-characterized biomolecular networks or pathways. The dynamics of some of these smaller pathways, like the MAPK signaling and Ras signaling pathways, have been described under various cellular conditions [7-9]. The EGFR signaling cascade has come under increased attention following observations of increased levels of EGFR gene expression in cancers of the lung, colon, endometrium, and other areas with significant epithelial tissue. A significant portion of that interest has been toward identification of highly conserved signaling modules or smaller pathways, which play a fundamental role in maintaining the robustness of the overall EGFR signaling network, and therefore, key to the persistent growth and development of tumour cells [3].

Recent advances in the development of new programming languages to describe parallel computer systems has proven beneficial toward overcoming the challenge of describing highly complex and parallel biological systems [10,11]. One particular programming framework known as the stochastic pi-calculus has allowed a wide range of biological systems to be modeled and simulated. The formal language of pi-calculus, originally developed for describing concurrent computational systems, specifies a framework to model large biological systems incrementally, intuitively building from simpler models of subsystems [12,13]. In pi-calculus, interaction between multiple biological processes can be described as synchronous transmission of messages between the processes. We can also represent biomolecules or complexes as processes, which allows transmissions to model chemical interactions or modifications of these molecules. This view concentrates on the components, their states, and their reactions and may lead to significant succinctness in representation of the model [14]. The underlying semantics of these models incorporates stochastic behaviour through association of interaction rates with channels and by setting the delays between reactions stochastically according to these rates [15]. A stochastic pi-calculus model can be efficiently simulated in a rigorous way that is faithful to chemical interactions. This enables us to perform *in silico *experimentation, and provide guidance for future experimentation. In addition, this modelling approach may be amenable to detailed analysis of the stochastic behavior of a network using model checking (cf [16]).

Previous studies of the EGFR signaling pathway have combined mathematical modeling and quantitative experimentation. These studies revealed insights into the functionalities of the pathway [6,17] and feedback loops [18]. By using experimental results to formulate and validate computational models, intrinsic behaviour of known EGFR modules can be explained at the system level. For instance, observations from kinase assays and computational simulations indicate that the MAPK cascade is robust and its signal response is quite insensitive to transient stimuli or low reaction rates [18,19]. Simulation of deterministic and non-deterministic models of the EGFR signaling cascade has demonstrated that signal responses to EGF stimulation becomes much more sustained as we move downstream in the cascade [20,21]. The attenuation of signal response is also found to become more insensitive to varying levels of EGF stimulation as we move closer to the ERK protein at the end of the cascade [21]. More recent studies using control analysis have suggested that only a small proportion of reactions substantially control signaling [6,22]. In particular, the Raf protein, an oncogene, was highlighted as an important mediator in the signal response of phosphorylated ERK, but little is known about the mechanism by which this control is exerted.

The ability of the EGFR signaling cascade, as with many other receptor tyrosine kinase signaling systems, to maintain a reproducible output despite input variations can be attributed to the module diversity and redundancy that allows for compensatory functioning in case of component failure [23]. It is this robustness feature of EGFR signaling cascades which enables signaling in tumour cells to be so resistant to anti-cancer therapeutics. But the high degree of reliance by the tumours on specific oncogenes or hub proteins in the signaling network gives us hope in identifying fragile sites in the network which may be possible targets for new drug therapies.

Here we present a modularized model of the EGFR signaling cascade within a single cell in the pi-calculus framework. Our model is adapted from previous mathematical models describing the same pathway [17,21]. Modules are not related to compartments in the cell and serve to emphasize the cause and effect relationships between different parts of the model. Compartments in the cell are indirectly represented in the model through their effect on reaction rates, as is also the case in the original mathematical models. Initial analysis shows that our model reproduces previous results. We further analyze this model by simulation under knock-out perturbations. These tests confirm the robustness of the upstream parts of the signalling pathway to perturbation; however, they also show that downstream parts of the pathway are less resistant to perturbations. Analyses under multiple simultaneous inhibitions reconfirm this relative robustness of up-stream modules and sensitivity of down-stream modules. Further investigation identifies positive feedback loops that help maintain stability of the signal under perturbations and negative feedback loops that help attenuate the signal and preserve its capacity over time. The analysis suggests a modular view of the signal. We suggest an abstract model concentrating on interfaces between the modules, which reproduces important behaviors of the pathway. Analysis of the abstract model confirms the importance of redundancies in upstream modules to preserve system robustness.

## Methods

### Model construction

The original kinetic model of the EGF-induced signaling pathway [6,21] was updated and implemented in the stochastic pi-calculus. The mathematical models in [6,21] describe the dynamics of this pathway in terms of kinetic equations which were converted into ordinary differential equations [21,24]. Our model in the stochastic pi-calculus (Additional File 1) contains kinetic parameters (e.g., rate constants of association and dissociation) and state variables (e.g., the number of molecules of a particular species or component). Second-order kinetics is used to describe enzyme-substrate binding, and also association/dissociation of EGF and signaling proteins to their receptors. The appropriate first- or second-order kinetics is used to describe each enzyme activity for a particular species (e.g. EGFR auto-phosphorylation and formation of ERK-PP). In the stochastic pi-calculus model, the dynamics of a component is described by the inputs and outputs of a process acting on communication channels. Once these processes are defined, communication channels are also defined with the communication rates associated with them. These rates are used to calculate the probabilities of all reaction events that happen in the biological system. The translation of the pi-calculus model to a continuous time Markov chain is standard [12]. We assume that the association of the different adaptive molecules is competitive, and binding of signaling molecules protects the corresponding phosphotyrosine residues from phosphatases. An EGF stimulus of 50 ng/ml is assumed to be enough to saturate all 50,000 EGF receptors of a HeLa cell. Further assumptions and estimation of parameters are described in the literature [6,21].

### Modularization and perturbation of the model

**Figure 1:**
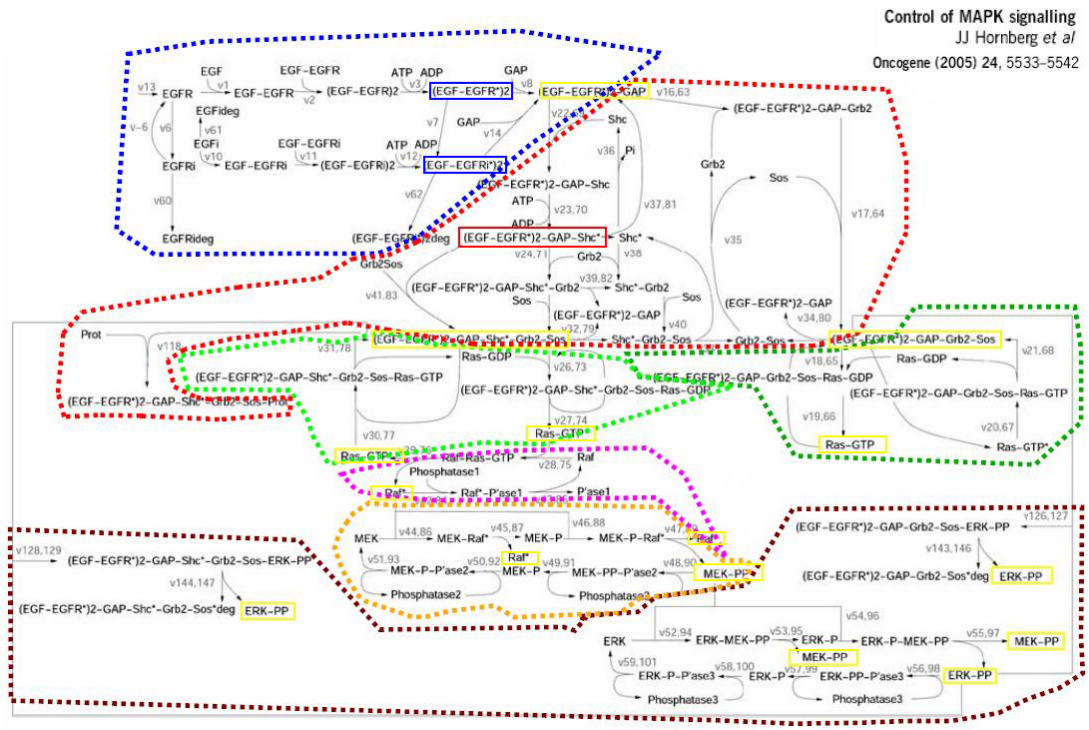
**The biochemical map of reactions involved in the EGFR signalling pathways **[6]. Reactants are connected by arrows that indicate interaction (and consumption) with other reactants. Each reaction has a number depicted in grey, and those with a second number refer to the reaction of internalized species. For example, reaction v1 binds together an EGF and an EGFR molecule; reaction v13 produces EGFR molecules. Each reaction can be also reversed. Dotted coloured lines indicate the boundaries of seven signaling modules: EGF (blue), Grb2 (red), Ras Shc-dependent (light green on the left), Ras Shc-independent (dark green on the right), Raf (pink), MEK (yellow), and ERK (maroon). The interface molecules which interact between modules are highlighted in yellow boxes.

The original model by Schoeberl *et. al*. [21] is modularized according to the location of oncogenes and components identified as important to the control of the EGFR signaling cascade [3,6,20,23]. Specifically, these protein components define most of the interface regions of the modules. Figure 1 depicts the substances and interactions covered by the model and illustrates the boundaries of the modules. Boundaries of modules are defined so that a small number of reactions cross between modules. We seek a partition of the model to components so that the molecules of one component do not interact with molecules of other components unless it is an interface of the component. We call such a region, where control mechanisms are contained within the boundaries of the region, a *signaling module*. Signaling modules are important for modularization of the EGFR signaling pathway as well as more generally for reuse and integration of modules in larger system models. Perturbations to the model were done by knocking-out reactions feeding in and out of the components highlighted in Figure 1 by setting the reaction rates to zero. Since we model inhibition of either association or dissociation of proteins and not both, we removed forward and reverse reaction rates independently. Forward reactions are specified by the direction of the arrows in Figure 1. Perturbation of sensitive reactions is achieved by multiplying the rate constants by a factor. For instance, 90% inhibition of the EGF-EGFR signaling complex's auto-phosphorylation process by tyrosine kinase inhibitors is modeled by multiplying the v3 forward reaction rate in the model by 0.1. The reaction rate is then converted into the communication rates defined on the communication channels.

### Stochastic pi-calculus

Similar to the procedure of modeling concurrent computational systems, biological systems can be described in the stochastic pi-calculus language. The behaviour of a molecule, such as a protein or a signaling complex, can be described as a process P, and actions describe possible interactions between molecules. Possible actions π include: (1) a delay action delay@r describes a change in the internal structure of a molecule with a delay whose rate is r. (2) an input action ?x(m) describes receiving a message from another molecule whose sending an output *m *on the same channel *x*. (3) an output action !x(n) describes sending a message to another molecule whose receiving an input *n *on same channel *x*. The specification do π_1_.P_1 _or ... or π_N_.P_N_, where π_i _stands for one of the three previous actions, represents the ability of a molecule to react in N different ways. The parallel composition P|Q represents the existence of molecules at the same time. By defining X(m) = P, we can define a molecule of type X parameterized by m. Each type of molecule will have its definition recorded in a constant environment E. For a more complete introduction to stochastic pi-calculus, we refer the reader to [12]. Essentially, the calculus allows a set of chemical reactions to be described in a modular fashion. This is achieved by splitting each reaction x into two complementary actions, a send !x and a receive ?x. The set of chemical reactions is then derived automatically by application of the rules of the calculus, which allow senders and receivers on the same channel to interact with each other. It is this ability to decompose a reaction into two parts that allows us to model the behaviour of individual components in a system. The pi-calculus approach allows a biological system to be programmed in a scalable manner, by allowing models of individual proteins to be directly composed to form subsystems, which can in turn be composed together to form larger systems. This allows new components to be added incrementally without having to re-wire the existing model, which also improves the usability of models. We stress the importance of pi-calculus for the ease of creation and simulation of perturbed models, as the parts of the model are directly related to the physical entities of the biological system. In essence, the behaviour of each substance in the system is described as a separate pi-calculus process. Thus, a mutation that relates to some substance is easily incorporated in the model by changing the corresponding pi-calculus process. Furthermore, the modular structure of the biological system and the pi-calculus model are in direct correspondence.

### Simulation and analysis

Our model was executed using the Stochastic Pi Machine (SPiM) simulator version 1.13. The stochastic pi-calculus code for executing the unperturbed model in SPiM was modified to run simulations on a perturbed EGFR signaling pathway. Each simulation of the EGFR model was carried over 3,600 time units, which is the equivalent of 60 minutes real time, and 100,000 sampling events took place over this time range. Observables generated by SPiM from the state variables were converted into absolute numbers of molecules for each species (each complex of different compounds was considered a unique species). Simulation of each perturbed model was carried over ten replicates and the number of molecules of ERK-PP was averaged over each time unit. The molecular profiles were plotted using the R-statistical platform, and a LOWESS method was applied to smooth the graphs.

In these simulations, we identify active signal response as the molecular profile of components in the signaling pathway when 50 ng/ml of EGF is added as a stimulus, and the inactive signal response as the molecular profile when no EGF is stimulating the system.

## Results

### Dependence of the signaling cascade response on EGF stimulation

**Figure 2:**
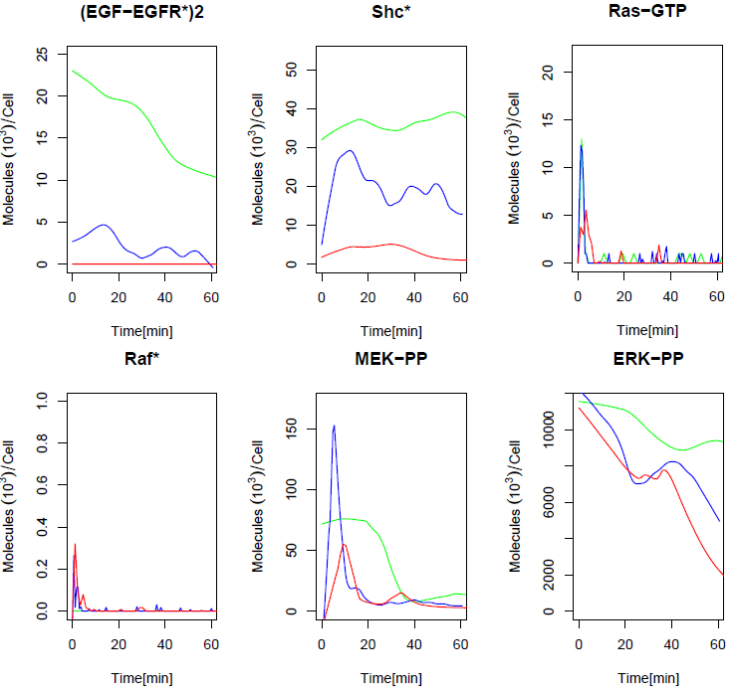
**Molecular profiles of molecular components at different EGF concentrations**. 50 ng/ml(green line), 25 ng/ml (blue line), and 0.5 ng/ml (red line). The (EGF-EGFR*)2 indicates the dimerized and autophosphorylated form of the EGF receptor. Shc* and Raf* is the phosphorylated form of cellular Shc and Raf. Ras-GTP is the formed complex of Ras and GTP. MEK-PP and ERK-PP are the doubly phosphorylated forms of MEK and ERK.

Our model of the EGFR signaling pathway is an updated version of previous models [17,21]. It is comprised of 148 molecular processes and 103 variable molecular species (Figure 1, Additional File 1). The signal cascade is initiated by induction of EGF to the system in the presence of other signaling molecules. We traced the dynamics of several important contributors to the signal cascade at varying levels of EGF stimulus, as shown in Figure 2. Results of these simulations are compared to simulations of previous models [17,21]. The close resemblance of simulations serves as a sanity check to the correctness of our translation from a mathematical to a computational model.

Analysis of these initial simulations reveals interesting features that hint to the results of later deeper analysis of the model. The activation of the EGF-receptors, in the dimerized and phosphorylated form, clearly depends on the concentration of EGF, responding with greater amplitude and earlier peaks. Although EGF concentration heavily affects membrane receptor activation, there is less concentration sensitivity as we trace down the signaling cascade. In particular, the amplitude and duration of the signal response in ERK-PP is hardly affected, even at low levels of stimulus. As suggested from the sharper and much shorter duration in the signal response of Ras-GTP and Raf-P, it is likely that the degree of activation in upstream components have little effect on the downstream signaling components. Following activation of Raf and Ras, there is considerable signal amplification occurring in the pathways leading to MEK and ERK activation. Even though the amplitude of the signal response does not seem to be significantly affected by the amount of EGF stimulation, the sustainability of the signal response in most components, including ERK-PP, seems to be attributed to the high EGF concentrations.

**Figure 3:**
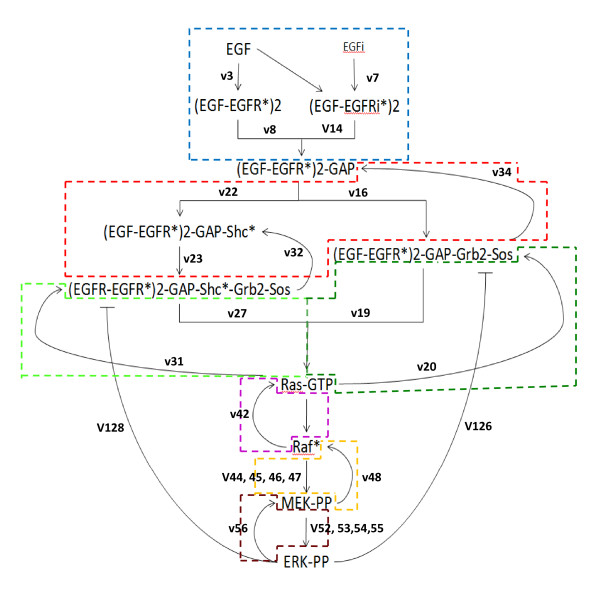
**Simplified graphical representation of the modules in the pathway with interface components**. Colored lines indicate the boundaries of the modules and the edge labels indicate reactions that are crucial to signaling between the molecular components.

We partition the model into functional modules based on the common biological concept of the importance of substances in the pathway. This partition is highlighted in Figure 1 and depicted in high level in Figure 3. Each signaling module contains signaling processes that lead to the activation of a key molecular complex and are observed as clusters in the signaling network. Therefore, the boundaries of the modules are indicated by molecules or nodes of high connectivity, where bottlenecks in the signaling network may occur. The signaling modules communicate with each other through the interface components at the boundaries, and the evolution of these components over time describes the EGF signal as it propagates down the pathway. We use the modules' boundaries as a guide to perturbations. We perturbed the EGFR signaling pathway model by systematically inhibiting each reaction completely and partially, as described below.

### Signaling network robustness and redundancy

**Figure 4:**
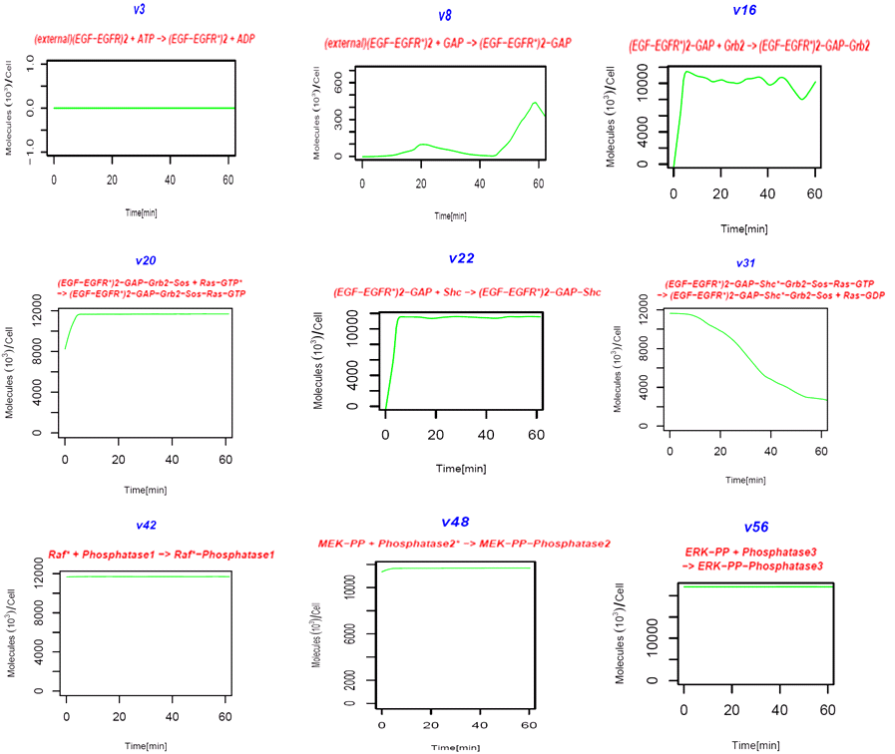
**Behavior of ERK-PP in response to complete inhibition of crucial reactions**. For each specific reaction that is inhibited, the molecular profile of ERK-PP is plotted over time.

An important characteristic of the EGFR signaling cascade is its ability to maintain output reproducibility, despite input variation or system perturbation. Design features, like network redundancy, may attribute to this functional robustness. Our first analysis consists of systematic inhibition of each reaction in the model separately. We include in Figure 4 simulation of the pathway under nine different knock-out perturbations. Additional simulations are given in Additional Files 2, 3, 4, 5, 6, 7, and 8. These simulations show regions of robustness in the signaling network. Evidence of robustness in the signaling network is particularly apparent in the Grb2 module, where insensitivity by the system to all single knock-outs is observed. This suggests for the presence of redundancy in the signaling network, which allows for signal sustainability even when parts of the signaling network are inactive. For instance, inhibition of a suggested target for kinase inhibitors, v23, did not have significant affect on signal transduction, except for the slight delay recorded in the activation of ERK. Note that the inhibition of either gateway reactions, v16 (leading to the Shc independent signaling pathway) and v22 (leading to the Shc dependent signaling pathway), do not inhibit ERK activation, but inhibition of both reactions does inhibit ERK activation. Although the EGF module may not contain as much redundancy as the Grb2 module, two pathways leading to the activation of internalized and external EGF receptors contribute to signal transduction. The simulations describe the sole dependence of ERK-PP signal response on the activation of external EGF receptors (v3), but the association of GAP with external receptors to form the EGFR signaling complex (v8) is not a necessary process for signal transduction. The inhibition of the v8 reaction does, however, drastically delay the activation of ERK and limit the amplitude of the signal response. Thus, the contribution of activated, internalized EGF receptors to EGF signaling remains unresolved.

### Greater sensitivity to perturbations in Raf, MEK and ERK modules

In the middle of the pathway, the Ras modules seem to maintain both fragile and robust network elements. Robustness of the two modules may be attributed to their extensive feedback mechanisms, and fragility may be due to the criticality of Ras-GTP for persistent signal transduction. Inhibition of v31 does not affect ERK from attaining maximal activation, but does affect the sustainability of its activated state (Figure 4). The observations suggest that the redundancy in the Ras Shc-independent pathway helps maintain greater network robustness than the Ras Shc-dependent pathway. But an interesting characteristic to be noted in the Ras Shc-independent pathway is how ERK-PP is continuously active when v20, a feedback mechanism, is removed - a sign of an inhibitory role Ras-GTP may play on ERK-PP. Significant, critical reactions are observed in the Raf, MEK and ERK modules. This may be attributed to the existence of redundant reactions, which play similar roles in propagating signal response and are not apparent in these modules. In particular, there is a chain of critical reactions spanning along the downstream modules, and completely inhibiting any of these reactions would halt signal transduction. Aside from the reactions important for the sustainability of signal transduction, simulations of our model also identify a number of reactions which may be crucial to the termination of the EGFR signal, like v42, v48 and v56. Reactions in the ERK module which do not affect ERK-PP evolution still play an important role in the inhibition of Sos-bound signaling complexes, and is identified as negative feedback. All this evidence suggests that each process in the downstream modules is involved critically in EGF signaling, therefore, contributing to a greater sensitivity to perturbations.

### The effect of single and multiple inhibitions on ERK signal

**Figure 5:**
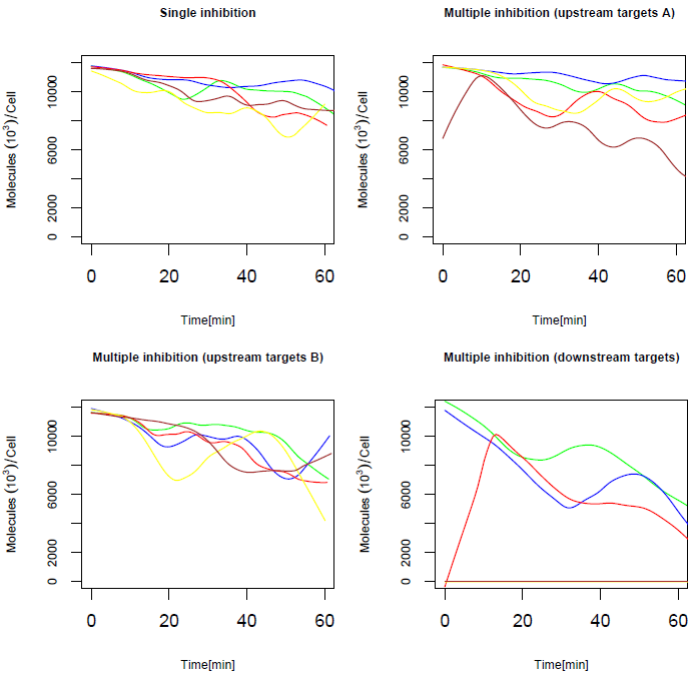
**Behavior of ERK-PP under various inhibitory conditions**. When the phosphorylation reaction of EGFR alone is targeted by kinase inhibitors (Single inhibition), when phosphorylation of EGFR and association of GAP are targeted (Multiple inhibitions: upstream targets A), when phosphorylation of EGFR and several other association reactions in the Grb2 module are targeted (Multiple inhibitions: upstream targets B), and when association reactions in the Ras Shc-dependent, Raf, MEK, and ERK modules are targeted (Multiple inhibition: downstream targets). Various levels of inhibition are represented by the green (10%), blue (30%), red (50%), yellow (70%) and brown (90%) lines.

Our next analysis is by simulation of the pathway under inhibition of multiple reactions (Figure 5). Reactions that caused sensitivity in the signaling network, as identified by the single knockout simulations, were exposed to varying levels of inhibition but never complete inhibition (knock-out). This simulates the action of hypothetical kinase and contact inhibitors on their respective phosphorylation and association reaction targets. The target for single inhibition is the phosphorylation of the EGF receptor (v3 and v7 reactions), which is known to be the site targeted by many tyrosine kinase inhibitors in common anticancer therapies. Inhibition activity at multiple sites (v3, v16, v23, and v25) in the upstream modules has a limited affect on ERK activation compared to single target inhibition. It is interesting that the combined reduction of EGF-receptor phosphorylation and GAP association rates have such little impact on ERK activation, even when complete inhibition eliminates EGF signaling. When multiple downstream signaling processes (v26, v28, v44, and v52) are targeted for inhibition, the ERK-PP signal response becomes much more sensitive to the levels of inhibition. The complete termination of EGF signaling for systems subjected to above 50% inhibition of downstream targets suggests that there exist a sensitive threshold for ERK activation. Lower levels of inhibition seem to affect the duration of the activated response, and increasing inhibition levels eventually delay the response and limit the activation maxima. Finally, there is evidence supporting the inhibitory effectiveness of combined inhibition at multiple target provides a much greater reduction in EGF signaling than 90% inhibition of a single phosphorylation reaction.

### Dependencies in Ras, Raf, and MEK signaling pathways

**Figure 6:**
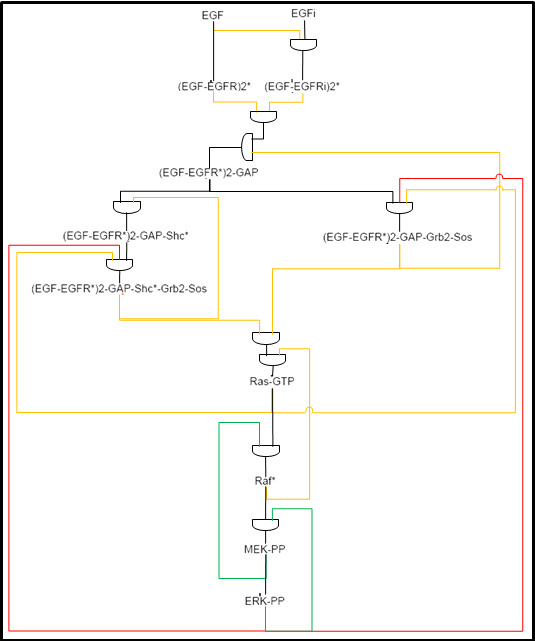
**Connectivity diagram of key components in the EGFR signaling pathway**. Each gate represents a series of reactions resulting in the production of the substance connected to the curved side. The substances connected to the flat side of a gate are the inputs. The initial inputs are set by the state of EGF species (internal and external). The connections show all paths between key components in the EGFR signaling pathway. Red connections indicate inhibition and all others activation. A connection is colored black or green if inactivation of the input disables activation of the output. Disconnection of **one **of the substances in orange or red does not affect activity of the output. The diagram clearly shows which paths carry signal that do not affect the overall activation of the component (red and orange wires), and which paths carry signals that are necessary to activate the component (green and black wires).

As a result of our simulations under extensive perturbation of the computational model, we can generate dependence relationships between key components in the EGFR signaling pathway. We exhaustively examined all conditions across the EGFR signaling pathway where components achieve active and inactive states. Activity/inactivity values for the molecular activities were compiled for each key component in order to map dependency of activity between these components. The resulting dependencies are summarized in Figure 6. The diagram includes all the interface components from Figure 1; interfaces are connected if one is the input to a module and the other is the output of a module or if the connection between them in Figure 1 is between the modules. That is, two interface components are connected in this diagram if they are connected in the full model in the same module or the connection between them does not pass other interfaces. We try to simplify the connection between these interfaces by concentrating only on their activity/inactivity state and deduce whether a component is required for the activation of another component or not (Additional Files 9, 10, 11, 12, 13, 14, 15, 16, and 17). A molecular component is defined as active if its observed profile corresponds to a profile of ERK-PP, where there are at least 5000 molecules for a period of 60 minutes. We inhibit specific reactions (column headings of Additional Files 9, 10, 11, 12, 13, 14, 15, 16, and 17) to generate the various knock-out conditions needed for inferring which connections are required or not. A connection is colored black or green if inactivation of the input disables activation of the output. Inactivation of **one **of the substances in orange or red does not affect activity of the output. In the case that a substance has only orange connections then one is required for activation but not both. The red connections denote inhibition and all others activation. Therefore, this representation of the EGFR signaling pathway provides a summary of the key mechanisms controlling signal transduction between components, and also suggests an intuitive way of understanding how the activation or inactivation of each component can affect the whole system. This representation captures the relations between substances but is not intended to be perceived as purely Boolean. We stress that deactivation of a red/orange input may still affect the amplitude or duration of activity of the signal, however, it remains active.

From the connectivity diagram in Figure 6 we can deduce the type of control mechanisms affecting each component. For instance, both Raf* and ERK-PP are required to the activation of MEK PP. The loop in the signal transduction from MEK-PP to ERK-PP and then back to MEK-PP, all of which are necessary for activation, highlights a critical positive feedback mechanism (green wires in Figure 6). Another instance of a positive feedback is identified by the connection from Raf* to Ras-GTP. Negative feedback mechanisms (red connections in Figure 6) are identified, whereby a signal from an activated component is a deactivating signal, as is the case with the two outputs from activated ERK. Where there are two orange connections entering a component then one signal is sufficient for activation. Our simulations reveal that Ras-GTP can be activated as long as there is a signal from either Sos signaling complexes (Shc dependent or Shc independent). Similarly, one of the internalized and externalized versions of the EGF receptors is sufficient for the activation of (EGF-EGFR*)2-GAP. When a component integrates required (black/green) and optional (red/orange) inputs then removing one of the optional inputs does not affect the overall activity of the component. For example, the activation of (EGF-EGFR*)2-GAP-Shc*-Grb2-Sos requires an activating signal from (EGF-EGFR*)2-GAP-Shc*, but signals from Ras-GTP and no signal from ERK-PP are optional for activation.

The connectivity diagram is justified by pi-calculus (SPiM) simulations. We have also created a smaller stochastic pi-calculus model that includes only these key molecular components and their connectivity as presented in Figure 6. This smaller model is obtained by replacing edges or sections of the pathway (Figure 1) that connect nodes in the diagram (Figure 6) by a single reaction. The rates limiting reaction between two interface components were chosen to set reaction rates for edges in the diagram. For instance, since the auto-phosphorylation reaction (v3) of the EGF-EGFR complex is rate limiting for the pathway between EGF to (EGF-EGFR)2*, its rate is used for the single reaction that transforms EGF to (EGF-EGFR)2* in the smaller model. The diagram only describes the key signaling pathways between the interface components of the modules, therefore, many short redundancies within the modules were abstracted away. In the implementation of the smaller pi-calculus model, parts of the pathway that are not represented in the diagram were removed through complete inhibition. Initial conditions for simulating the smaller model were identical to those used for the full pi-calculus model. A consistent signal response could not be generated using just the connectivity diagram. Simulation of the reduced pi-calculus model showed attenuation of the signal response in the Grb module, specifically, we observed no activation signal at the (EGF- EGFR*)2-GAP-Shc*-Grb2-Sos and (EGF- EGFR*)2-GAP-Grb2-Sos complexes (Additional File 18). When we provide an activation signal for those two complexes and carried on with the rest of the simulation downstream in the pathway, activation of ERK-PP is achieved (Additional File 19). As seen in the diagram, the abstracted version of the Grb module is represented by two edges from (EGF-EGFR*)2-GAP to EGF-EGFR*)2-GAP-Shc*-Grb2-Sos and (EGF-EGFR*)2-GAP-Grb2-Sos. However, when we substitute this abstract Grb module with the Grb module from the full model (with all the redundancies), activation of ERK-PP is also achieved. That is, the abstract model with one concrete module generates a behavior comparable to that of the full model. This suggests that the removal of redundant pathways in the Grb module prevented our abstract model from simulating signal.

## Discussion

This work presents a computational model of the EGFR signaling pathway. The model, written in the stochastic pi-calculus language, represents our current understanding of EGFR signaling dynamics and the contribution of important signaling components to this pathway. Execution of the model allowed us to simulate and observe signal transduction through the pathway under various conditions. Some simulations match the experimental observations and increase our confidence in the model and its consistency with previous models [24,27,21]. Other simulations enable us to further analyze and hypothesize on the roles of different components in the pathway. Our aim was to identify and analyze the control mechanisms associated with key components. We simulated single knock-outs and multiple inhibitions of reactions. Our choice for inhibitions is guided by a partition of the model into behavioral modules that are inspired by the biological understanding of importance of the different components in the pathway. We could identify differences in network robustness and inhibition sensitivity between the different parts of the signal network.

The insights gained from simulation and analysis of the kinetic behaviour of the EGFR signaling pathway helps us understand not only the terminal signal response to EGF stimulus, but also the response at various levels of the signaling cascade. The analysis suggests that there is high sensitivity in upstream components to variations in transient EGF stimulus, but this sensitivity does not translate to differential signal response in downstream components. Specifically, sensitivity analysis reveals greater sensitivity in the EGF binding reaction and the Raf dephosphorylation reaction [22]. In addition to this, the significant amplification of the EGF signal reported by the profile trace from the upstream signaling components to downstream components, even at low EGF dosages, highlights the importance of binding kinetics to the formation of the EGFR signaling complex [17]. As suggested by previous models [20,21], and also apparent in our simulations, attenuation of signal response occurs after an apparent reduction in signal duration caused by decreasing levels of EGF stimulus. It is clear from the results of the simulations at varying levels of EGF stimulus that in order to accurately predict the response to EGF stimulus, we need to break down our EGFR model and understand the functionality of intermediate signaling components.

Partitioning of the EGFR signaling network into modules is not a trivial task, but it leads to a simpler model of the signaling cascade which offers clarity and computational tractability [25]. Identification of modules based on the clustering of network components and localization of signal control mechanisms is presented as an approach to attaining a simplified version of the EGFR signaling model. The observed modularity of the EGFR signaling pathway has offered us an insight into the reuse of shared pathways in the cellular system. We know that many biological networks have features suggesting that they have modularity, defined by the separability of its design into independently functioning units [26]. Furthermore, these network modules show reuse in many different parts of the system, and allow for the construction of extremely complex systems by using simple building blocks [27]. The common signaling modules identified in our model could be used as building blocks for models of other systems or more complex networks. Reuse of these modules in future models would be very simple, because the stochastic pi-calculus implementation of our model allows for the code to be partitioned easily and integrated with other models implemented in the same language.

By comparing the results of the simulations in terms of signaling modules, we further verify differences between modules based on network characteristics, like redundancy and robustness to perturbations. Some of these characteristics, such as the dependence of ERK-PP signal response on the activation of EGF receptors in the EGF module, may be an indication of why contact and tyrosine kinase inhibitors continue to be an elective means of preventing EGFR signaling [28]. Our knock-out simulations confirmed the network robustness of the Grb2 module, which can be attributed to the forking of the activation signal into both Shc-dependent and Shc-independent pathways. Although signal response primarily proceeds through the Shc-dependent pathway [22], as shown by lack of signal attenuation when reactions feeding into the Shc-independent Ras module are knocked out, significant redundancy and feedback mechanisms allow for signal retention when either pathway fails. Also, another characteristic, present particularly in the Grb2 module and may be an explanation for the sustainability of signal transduction in the face of partial system failure, is the presence of numerous of reservoirs of Shc, Grb and Sos compounds that are involved in a number of reactions feeding into interface components. It is then interesting to note that simulations conducted with other network models show the distribution of Sos to be higher in regions of the network with active signal transduction compared to regions disrupted by mutations [29]. Such a result demonstrates the signaling network's ability to adapt and alter signaling component profiles when under unfavorable conditions. In contrast, the lack of network redundancy and the lack of reservoirs of important compounds acting on various parts of downstream signaling modules results in greater network fragility for those downstream modules. This places extra responsibility on the interface components (Ras-GTP, Raf-P, and MEK) to maintain signal transduction in the presence of an activating signal. We provide evidence which shows that while the activity of Raf is in strong control of all characteristics of the transient profile in ERK phosphorylation [6], the combined activity of other downstream components may also have strong control over ERK phosphorylation. Our simulation results suggest that extra sensitivity in the downstream regions of the EGFR signaling pathway may be exploited for the purpose of inhibiting EGFR signaling in tumour cells.

The ability to perturb a biological system without any cost, except for those that are computational, truly attests to the important practicality of conducting *in silico *experimentations on computational models. More importantly, computational models have been widely used in the fight against numerous detrimental diseases by helping to determine possible targets for drug therapies. Interestingly, through our tests to determine the effectiveness of targeting multiple inhibition sites as a means of combination therapy, we discovered a higher level of system sensitivity to targets in the downstream modules (Raf, MEK and ERK) as opposed to targets in the upstream modules (EGF and Grb2). Specifically, we did not detect any evidence of significant attenuation of EGFR signaling when phosphorylation reactions in the upstream modules are targeted for partial inhibition, contradicting the results of analysis done on other mathematical models [24]. Since high sensitivity to reaction rates in the upstream modules were reported [6], it would not be surprising that discrepancies in these results are attributed to differences in reactions rates and initial conditions chosen by the different models. Although our results may suggest that the combined inhibitory targeting of multiple downstream reactions in the EGFR signaling pathway could be an effective means of halting EGFR signaling in tumour cells, utilizing these sites as an actual drug target would be a daunting challenge due to lack of specificity, since these reactions appear in many important cellular processes.

As a means of compiling our observations of the various states that the components in the EGFR signaling pathway achieved under different conditions, and as a way of demonstrating model abstraction with the aid of models in the pi-calculus language, we produced a simplified connectivity diagram (Figure 6). The diagram depicts signal transduction through the EGFR signaling pathway abstractly. We summarize the control mechanisms determining the activation state of an interface component. From this graphical representation, we can easily identify relationships in the EGFR signaling pathway that are essential and those that are optional. Essential positive feedback mechanisms shown in the diagram have been well studied and their ability to produce an actively maintained 'memory' of transient inductive stimulus in the MAPK signaling cascade has been characterized [18]. While the negative feedback loops stemming from ERK-PP are shown to bring about adaptation and robustness to parameter variations, they can also induce dampened or sustained oscillations whenever activation attains certain threshold strength [4]. The diagram also supports the notion that while single inhibition may lead to loss of signal output, dual inhibition may lead to partial restore of the lost function [22]. Alternative methods of graphically describing the signaling relationships involving the EGFR pathway have been proposed [30,31], and although they seek to depict the enormous amount of information to be conveyed to each component in the network and describe the high cross-connectivity in the pathway, scalability becomes an issue with these methods and it would be difficult to systematically translate their graphical model to automata. One can also argue that our diagram makes over-simplifications toward the discreteness of the signaling system, and non-apparent relationships between signaling components may be lost in the model abstraction. In the case of the Grb module, removal of its redundant pathways in our pi-calculus simulations shows that signal transduction cannot occur without those redundancies. However, as exhibited by simulation of the abstract model with the full Grb module reproducing signal transduction, the rest of the abstract model does capture essential parts of the signalling pathway. This is why we present the connectivity diagram here as a summarization of the dynamics in the EGFR signaling pathway. Since the EGFR signaling pathway can be described as a formal system, there is great opportunity for other complex biological signaling pathways to be modeled and validated using formal verification methods [32].

In order to perform our analysis we have chosen to use the SPiM simulator, which is based on the pi-calculus formalism. There is a wide range of existing tools based on process calculi, such as BetaWorkbench [33], Bio-Pepa [34], and Cellucidate [35], which all use Continuous Time Markov Chains as the underlying computational model. Each tool has its own distinctive characteristics and advantages; for example, Cellucidate is a rule-based formalism, which directly models the reactions between components as opposed to the behaviour of the individual components themselves. Rule-based modeling gives greater flexibility in describing the reactions of a biological system. Bio-Pepa is, likewise, a reaction-based formalism in which the reactants and products can be described separately. BetaWorkbench relies on an individual-based formalism, where each biological entity is encapsulated inside a boundary and local processes inside the boundary are used to model changes in state. This extra layer gives more modeling flexibility but at the expense of additional complexity. In general, expert modelers could have used any one of these languages to create the EGFR model presented here. Our choice of SPiM was motivated by our interest in perturbed versions of the same model. Using SPiM, we were able to automate thousands of model simulations using a simple command-line script. In addition, SPiM's individual-based formalism, simplified language primitives, and its support for a graphical representation of models, simplified the creation and manipulation of this model. In particular, the one-to-one correspondence between the graphical notation and program code allows both graphics and text to be used interchangeably. Process calculi are rapidly establishing themselves as computational formalisms of choice for modeling molecular interactions. Stochastic or hybrid versions of Petri Nets [36] can be viewed as a reaction-based modeling formalism that supports a range of powerful analysis techniques, though its core language is not as rich as process calculi. StateCharts [37], on the other hand, provide an extremely rich language for modeling, but at present do not support exact stochastic simulation techniques and, like discrete versions of Petri Nets, are thus more appropriate to create abstract models of biological systems. For a review of several computational approaches and their adequacy for different kinds of models we refer the readers to [38].

## Conclusions

Through simulation of the EGFR signaling model under different perturbation conditions, we can discover regions of network robustness and fragility. The molecular profiles of the important signaling components presents evidence for the increasing insensitivity to low EGF stimulus as we move down the signaling pathway. In contrast, greater sensitivity is exhibited by the signaling system when downstream signaling modules are perturbed. There seems to be significant redundancy that is contributing to network robustness in Grb2 and Ras signaling modules. Further study of partial inhibition at the important reactions demonstrates that high signal attenuation is achieved when multiple sites in downstream signaling modules are targeted. By analyzing the behaviour of interface components as the signal passes through the modules, we are able to produce a mapping of signaling modules to simplified operations, and thereby, generating a connectivity diagram for the EGFR signaling pathway. From the diagram, we can see important feedback mechanisms, some of them crucial to the maintenance of activated response in ERK and could explain the irreversible maturation of tumour cells. As is shown, the results from these simulations of the EGFR signaling pathway can be used to help formulate hypotheses, regarding the dynamics of EGFR signaling, to be tested in the 'wet lab'. Furthermore, the experimental results can be used to eliminate spurious assumptions in the model and hence further verify it.

## Authors' contributions

DW, NP, and JF conceived and designed the computational simulations. AP and LC participated in crucial discussions. AP provided technical advice with designing the SPiM experiments and running the SPiM simulations. LC implemented an earlier version of the EGFR signaling pathway model, which served as a basis for the current modular version of the model that was implemented by AP. DW edited the model, performed simulations and analyzed the data. DW, NP, LC, and JF wrote the manuscript. AP contributed to the additional files. All authors read and approved the final manuscript.

## Supplementary Material

Additional file 1 **Stochastic pi-calculus model of the biochemical interactions involved in the EGFR signalling pathways**. The model is based on the reaction map of Figure 1. Essentially, each connected graph represents the set of possible states of a given protein, where each node represents a protein in a particular state. For example, in the top right there are three graphs for proteins Phase1, Phase2 and Phase3, where each of these proteins can be in two possible states, bound or free. Proteins can change their state by interacting with each other over shared channels, where each labelled edge represents an action that a protein can perform in order to change from its current state to a new state. For example, the protein Phase1 can become bound by interacting on channel phase1, and can then become free by interacting on channel p. The channel phase1 is parameterised by p, written phase1(p). This indicates that the protein becomes bound on p after the interaction takes place. In this figure we have grouped sets of proteins together into subsystems, denoted by rectangular boxes. For example, in the top right we have grouped the proteins Phase1, Phase2, Phase3, Raf, MEK and ERK into a cascade subsystem. All the channels inside a given subsystem are local to that subsystem, apart from those that are represented between boxes. For example, in the cascade subsystem the channels phase1, phase2 and phase3 are local and cannot interact with any of the other subsystems, while channels rasgtp and erkpp can interact with two other subsystems. This gives an overall schematic for the interactions between the various subsystems. We observe that the ERK subsystem can interact with the Grb2 subsystem on channel erkpp. Similarly, the EGF subsystem can interact with the Grb2 subsystem on channel complex, representing the EGF-EGFR'2-GAP complex. Finally, we observe that the RasGTP subsystem can interact with both the cascade and the Grb2 subsystem. Note that the interactions with RasGTP are parameterised by channels r, r' and rd, indicating that the bound RasGTP protein can unbind in three different ways, resulting in RasGTP, RasGTP' or RasGDP. A corresponding set of chemical reactions can be automatically generated for the entire system, but note that the translation does not preserve the modular description of the system. The model is available at http://research.microsoft.com/en-us/um/people/aphillip/egfr09/ and the SPiM simulator is available at http://research.microsoft.com/spim/ Click here for file

Additional file 2 Behaviour of ERK-PP in response to complete inhibition of key reactions in the EGF module. Click here for file

Additional file 3 Behaviour of ERK-PP in response to complete inhibition of key reactions in the Grb2 module. Click here for file

Additional file 4 Behaviour of ERK-PP in response to complete inhibition of key reactions in the Ras Shc-independent module. Click here for file

Additional file 5 Behaviour of ERK-PP in response to complete inhibition of key reactions in the Ras Shc-dependent module. Click here for file

Additional file 6 Behaviour of ERK-PP in response to complete inhibition of key reactions in the Raf module. Click here for file

Additional file 7 Behaviour of ERK-PP in response to complete inhibition of key reactions in the MEK module. Click here for file

Additional file 8 Behaviour of ERK-PP in response to complete inhibition of key reactions in the ERK module. Click here for file

Additional file 9 Boolean table for inferring logical inputs for (EGF-EGFRi)2*. Click here for file

Additional file 10 Boolean table for inferring logical inputs for (EGF-EGFR*)2-GAP. Click here for file

Additional file 11 Boolean table for inferring logical inputs for (EGF-EGFR*)2-GAP-Shc*. Click here for file

Additional file 12 Boolean table for inferring logical inputs for (EGF-EGFR*)2-GAP-Grb2-Sos. Click here for file

Additional file 13 Boolean table for inferring logical inputs for (EGF-EGFR*)2-GAP-Shc*-Grb2-Sos. Click here for file

Additional file 14 Boolean table for inferring logical inputs for Ras-GTP. Click here for file

Additional file 15 Boolean table for inferring logical inputs for Raf*. Click here for file

Additional file 16 Boolean table for inferring logical inputs for MEK-PP. Click here for file

Additional file 17 **Molecular profiles of interface components as a result of perturbations on two different input signals. **See highlighted rows in Additional Files 10, 11, 12, 13, 14. Click here for file

Additional file 18 Molecular profile of ERK-PP in response to signal from the EGF-EGFR complex as simulated by the abstract pi-calculus model. Click here for file

Additional file 19 Molecular profile of ERK-PP in response to signal from the EGF-EGFR complex as simulated by the abstract pi-calculus model, but without abstraction of the Grb module. Click here for file
