# Supplementary figures and images for: Computational modeling of the EGFR network elucidates control mechanisms regulating signal dynamics
Source: BMC Syst Biol. 2009 Dec 22;3:118. doi: 10.1186/1752-0509-3-118 (PMC2807436; doi:10.1186/1752-0509-3-118)

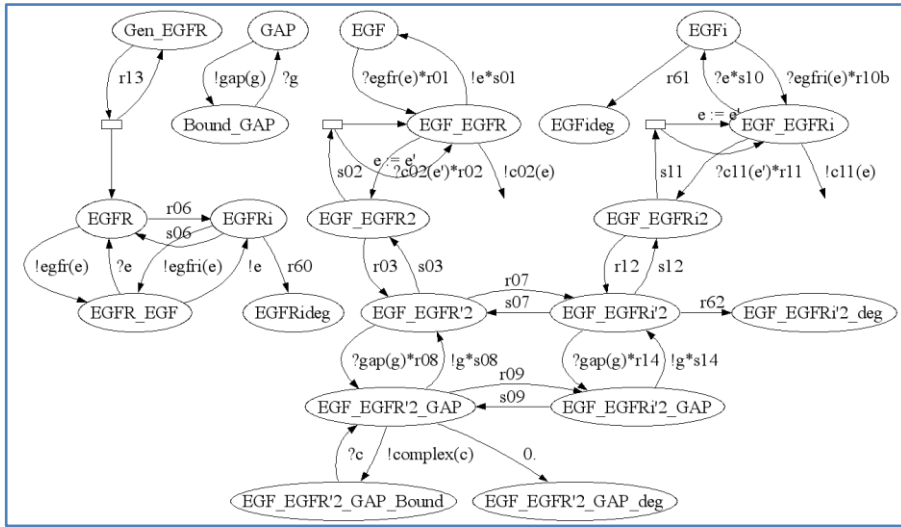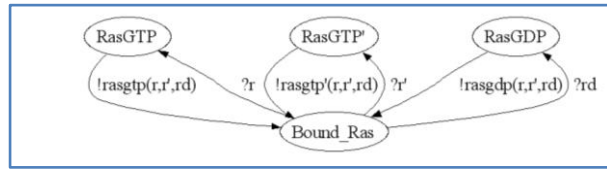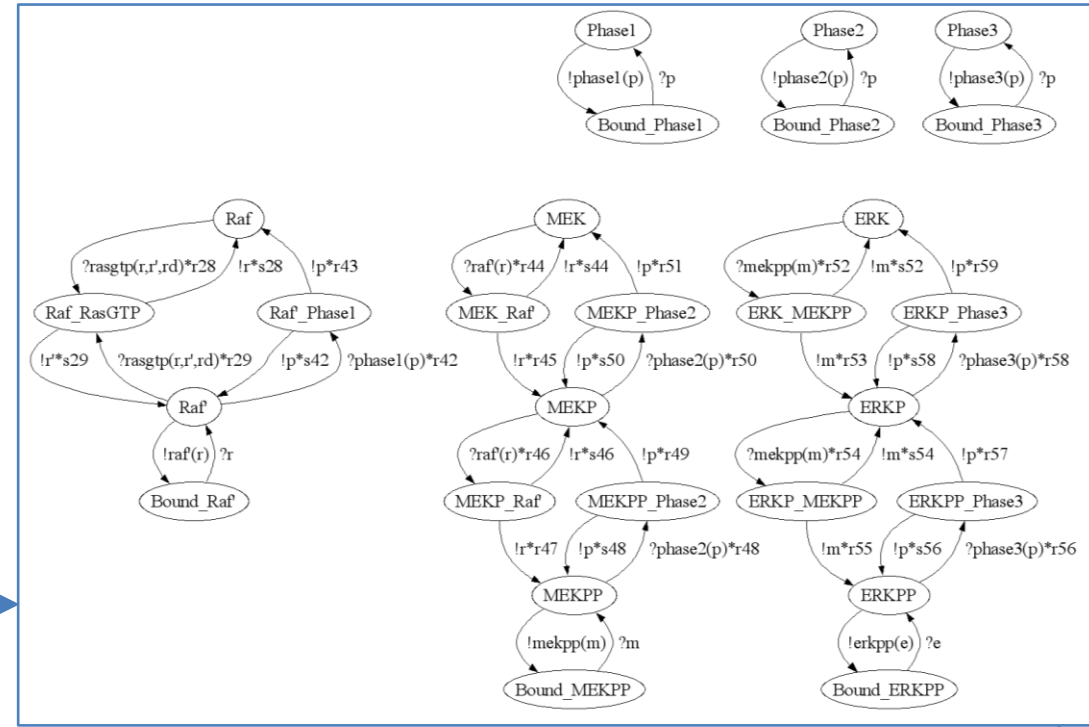

complex(c)

rasgtp(r,r',rd)

rasgtp(r,r',rd)  
rasgdp(r,r',rd)  
rasgtp'(r,r',rd)

erkpp(e)

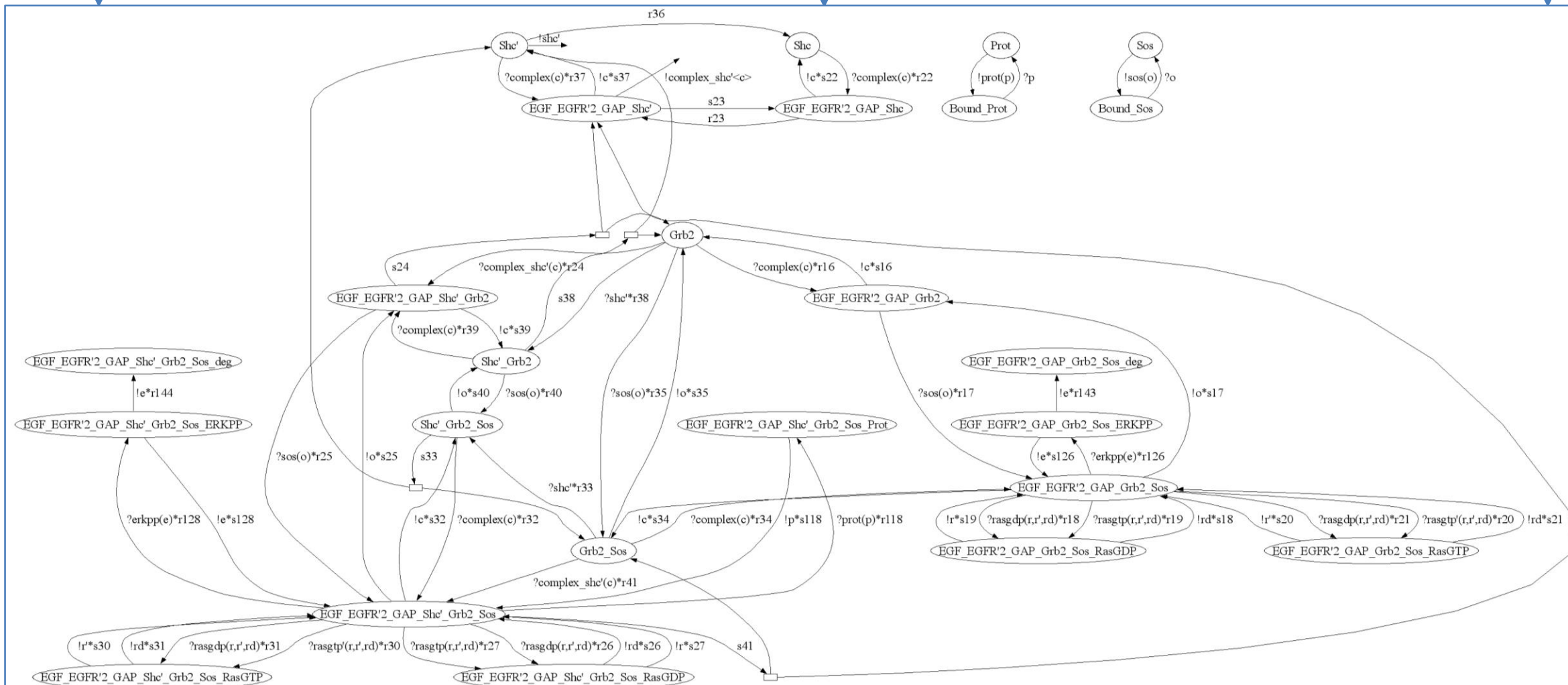

Supplement: Additional file 1 — Stochastic pi-calculus model of the biochemical interactions involved in the EGFR signalling pathways. The model is based on the reaction map of Figure 1. Essentially, each connected graph represents the set of possible states of a given protein, where each node represents a protein in a particular state. For example, in the top right there are three graphs for proteins Phase1, Phase2 and Phase3, where each of these proteins can be in two possible states, bound or free. Proteins can change their state by interacting with each other over shared channels, where each labelled edge represents an action that a protein can perform in order to change from its current state to a new state. For example, the protein Phase1 can become bound by interacting on channel phase1, and can then become free by interacting on channel p. The channel phase1 is parameterised by p, written phase1(p). This indicates that the protein becomes bound on p after the interaction takes place. In this figure we have grouped sets of proteins together into subsystems, denoted by rectangular boxes. For example, in the top right we have grouped the proteins Phase1, Phase2, Phase3, Raf, MEK and ERK into a cascade subsystem. All the channels inside a given subsystem are local to that subsystem, apart from those that are represented between boxes. For example, in the cascade subsystem the channels phase1, phase2 and phase3 are local and cannot interact with any of the other subsystems, while channels rasgtp and erkpp can interact with two other subsystems. This gives an overall schematic for the interactions between the various subsystems. We observe that the ERK subsystem can interact with the Grb2 subsystem on channel erkpp. Similarly, the EGF subsystem can interact with the Grb2 subsystem on channel complex, representing the EGF-EGFR'2-GAP complex. Finally, we observe that the RasGTP subsystem can interact with both the cascade and the Grb2 subsystem. Note that the interactions with RasGTP are parameter [file 1752-0509-3-118-S1.PDF]

v3\_v12

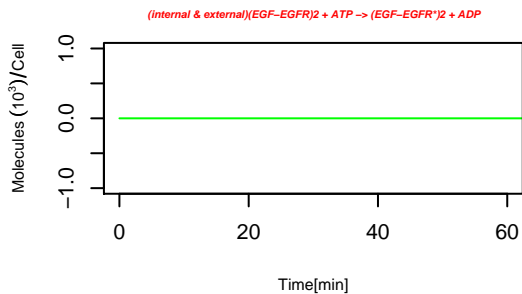

v3

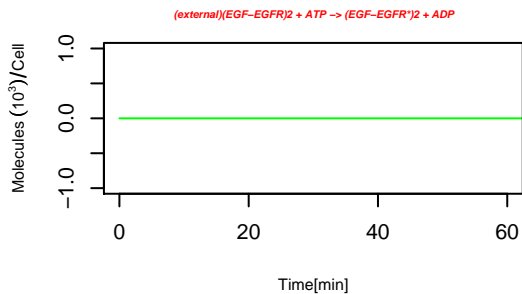

v7

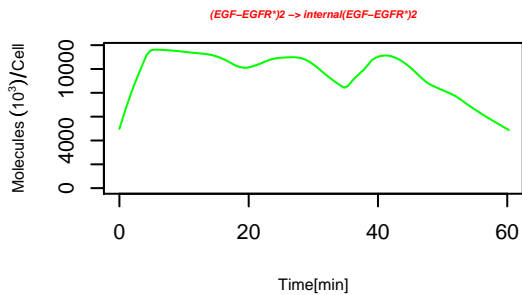

v62

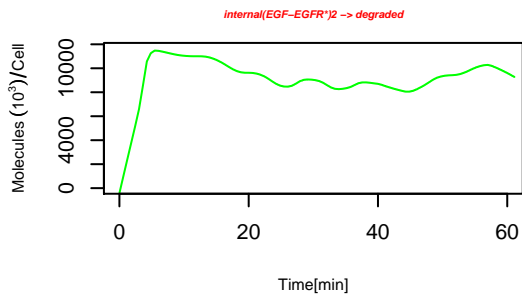

v8\_v14

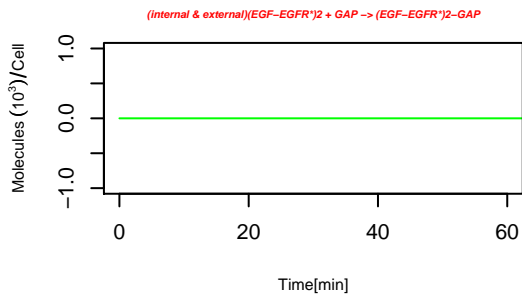

v8

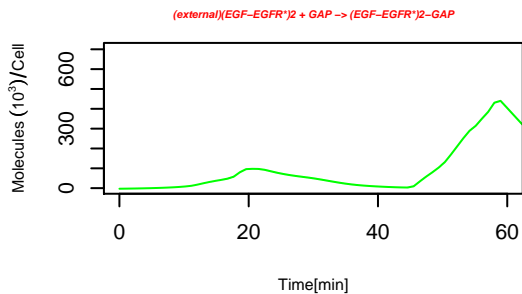

Supplement: Additional file 2 — Behaviour of ERK-PP in response to complete inhibition of key reactions in the EGF module. [file 1752-0509-3-118-S2.PDF]

v20

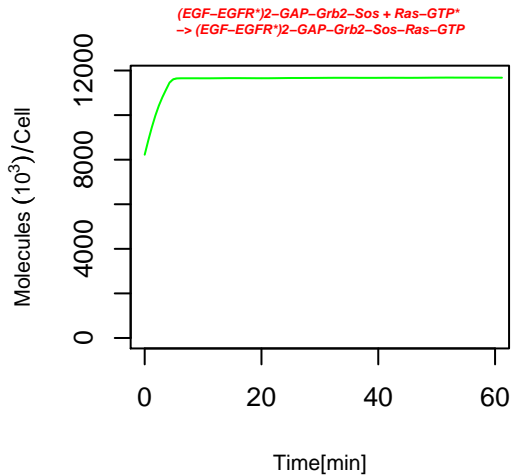

v18

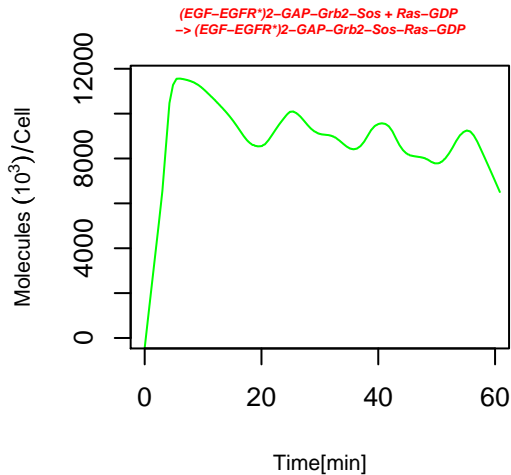

v21

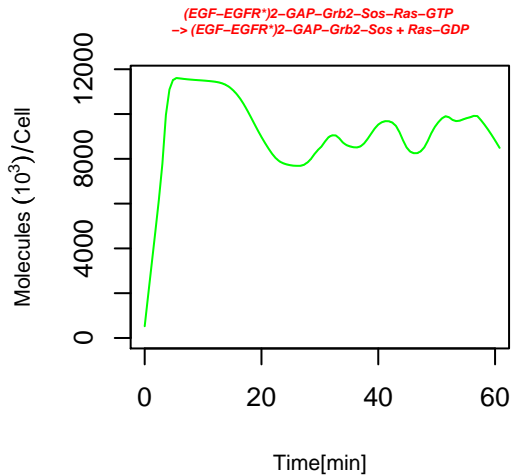

v19

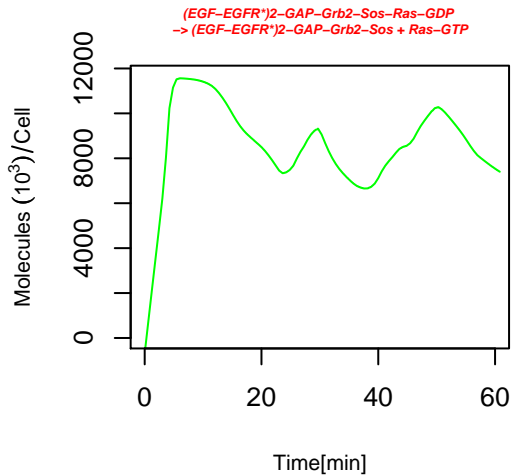

Supplement: Additional file 4 — Behaviour of ERK-PP in response to complete inhibition of key reactions in the Ras Shc-independent module. [file 1752-0509-3-118-S4.PDF]

**v30**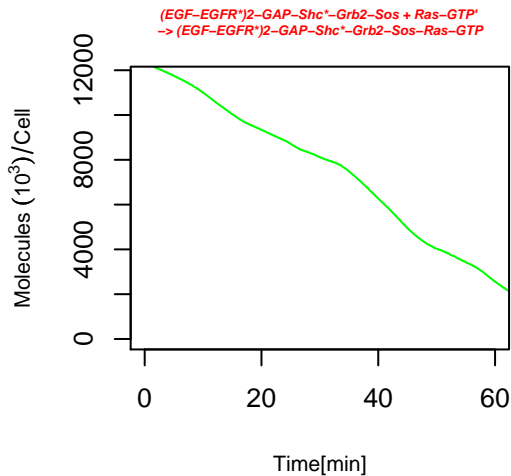**v31**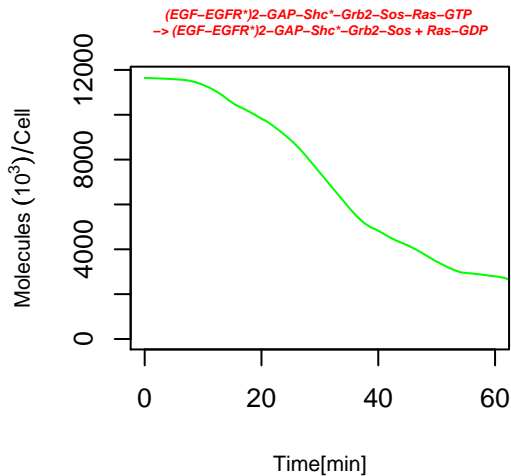**v26**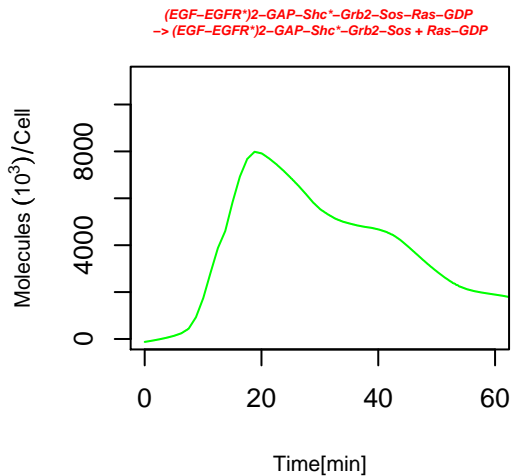**v27**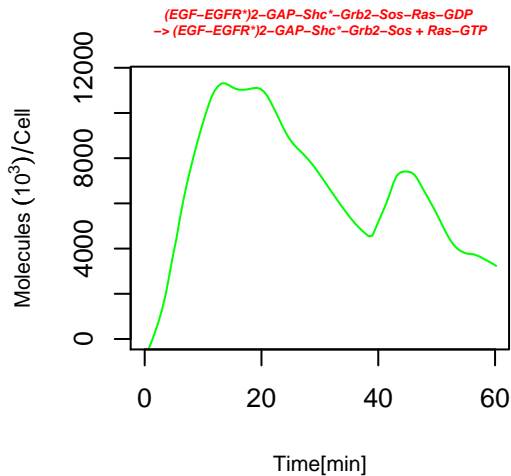

Supplement: Additional file 5 — Behaviour of ERK-PP in response to complete inhibition of key reactions in the Ras Shc-dependent module. [file 1752-0509-3-118-S5.PDF]

**v28**

*Raf + Ras-GTP -> Raf-Ras-GTP*

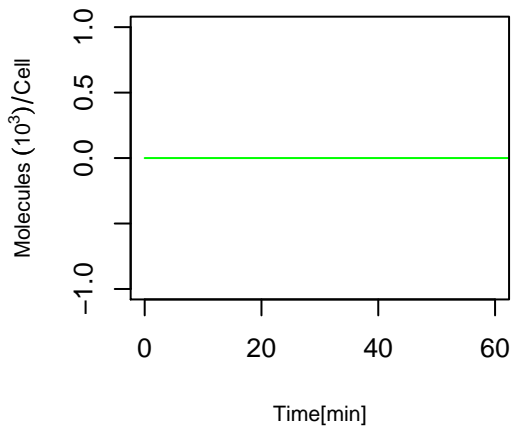

**v29**

*Raf-Ras-GTP -> Raf\* + Ras-GTP\**

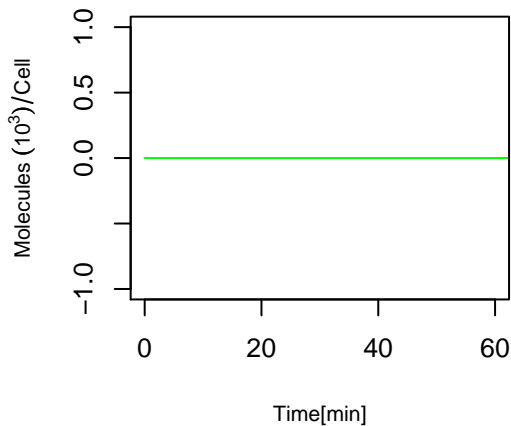

**v42**

*Raf\* + Phosphatase1 -> Raf\*-Phosphatase1*

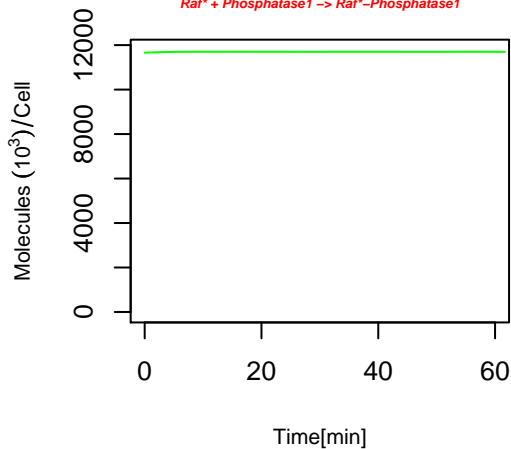

Supplement: Additional file 6 — Behaviour of ERK-PP in response to complete inhibition of key reactions in the Raf module. [file 1752-0509-3-118-S6.PDF]

**v52**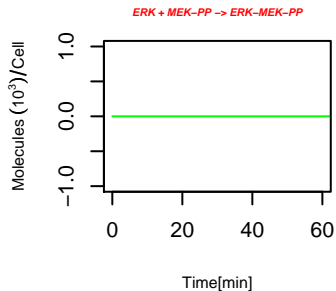**v54**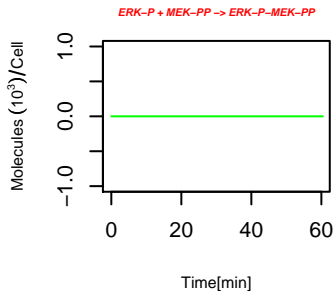**v53**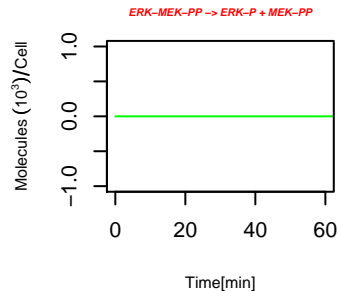**v55**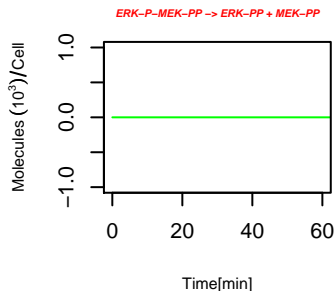**v56**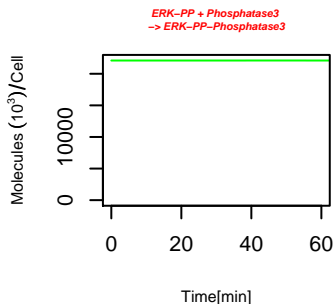**v126**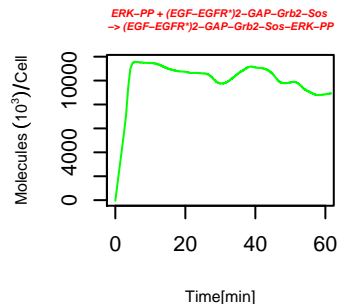**v128**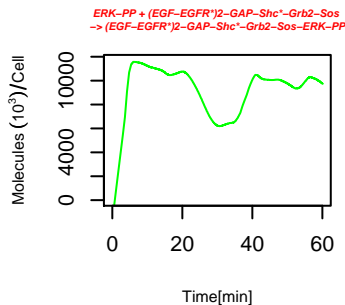**v144**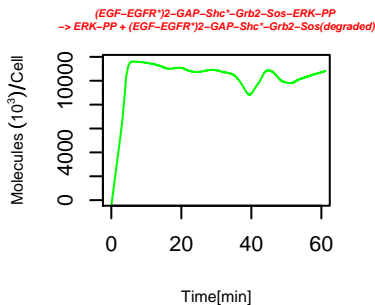**v143**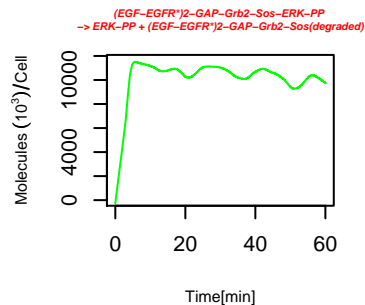

Supplement: Additional file 8 — Behaviour of ERK-PP in response to complete inhibition of key reactions in the ERK module. [file 1752-0509-3-118-S8.PDF]

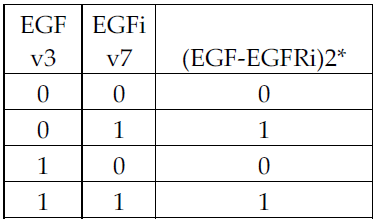

Supplement: Additional file 9 — Boolean table for inferring logical inputs for (EGF-EGFRi)2*. [file 1752-0509-3-118-S9.TIFF]

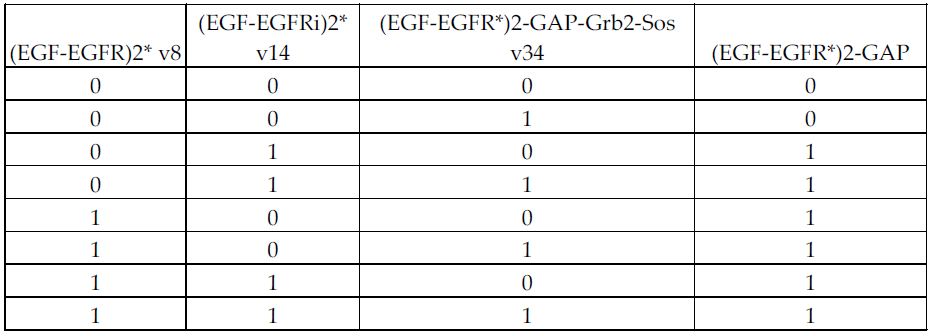

Supplement: Additional file 10 — Boolean table for inferring logical inputs for (EGF-EGFR*)2-GAP. [file 1752-0509-3-118-S10.TIFF]

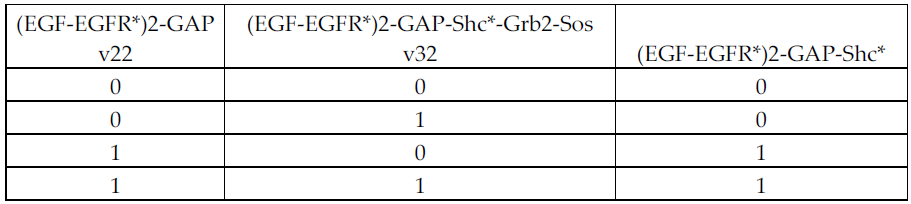

Supplement: Additional file 11 — Boolean table for inferring logical inputs for (EGF-EGFR*)2-GAP-Shc*. [file 1752-0509-3-118-S11.TIFF]

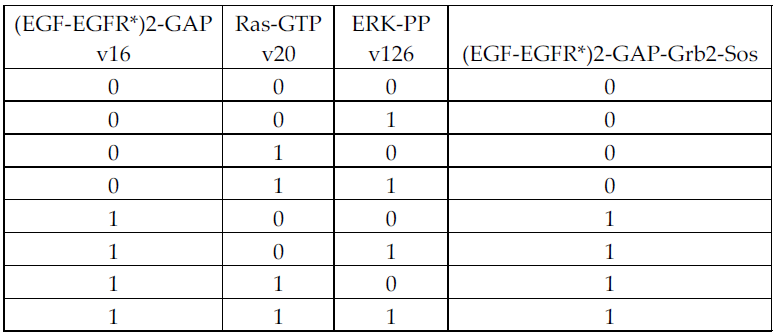

Supplement: Additional file 12 — Boolean table for inferring logical inputs for (EGF-EGFR*)2-GAP-Grb2-Sos. [file 1752-0509-3-118-S12.TIFF]

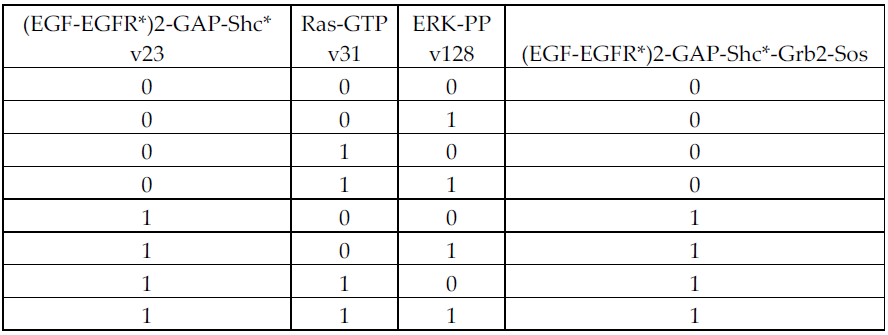

Supplement: Additional file 13 — Boolean table for inferring logical inputs for (EGF-EGFR*)2-GAP-Shc*-Grb2-Sos. [file 1752-0509-3-118-S13.TIFF]

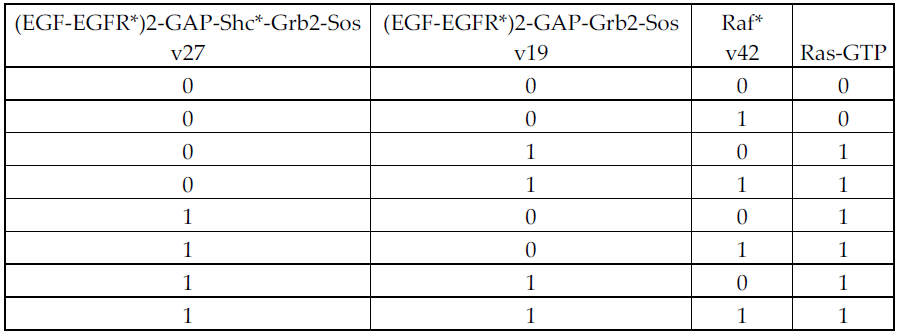

Supplement: Additional file 14 — Boolean table for inferring logical inputs for Ras-GTP. [file 1752-0509-3-118-S14.TIFF]

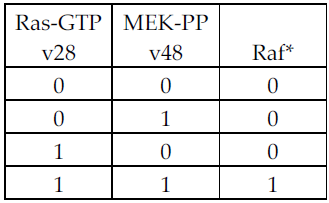

Supplement: Additional file 15 — Boolean table for inferring logical inputs for Raf*. [file 1752-0509-3-118-S15.TIFF]

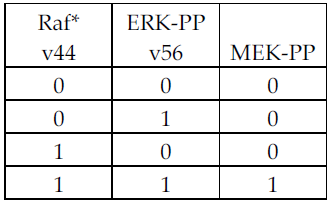

Supplement: Additional file 16 — Boolean table for inferring logical inputs for MEK-PP. [file 1752-0509-3-118-S16.TIFF]

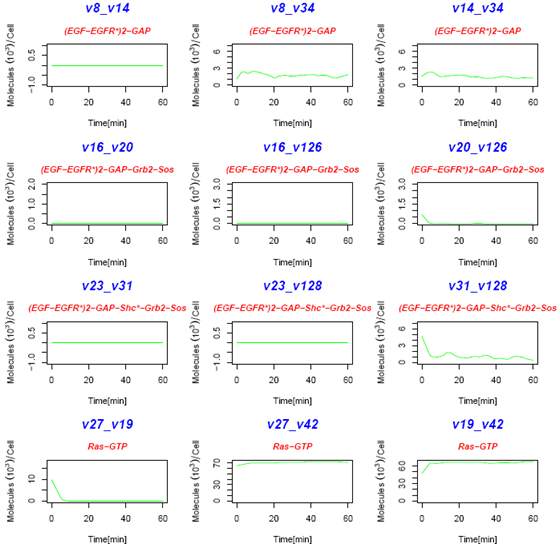

Supplement: Additional file 17 — Molecular profiles of interface components as a result of perturbations on two different input signals. See highlighted rows in Additional Files 10, 11, 12, 13, 14. [file 1752-0509-3-118-S17.TIFF]

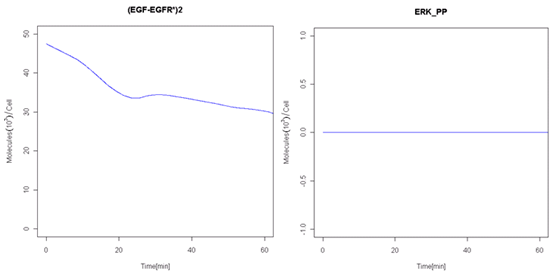

Supplement: Additional file 18 — Molecular profile of ERK-PP in response to signal from the EGF-EGFR complex as simulated by the abstract pi-calculus model. [file 1752-0509-3-118-S18.TIFF]

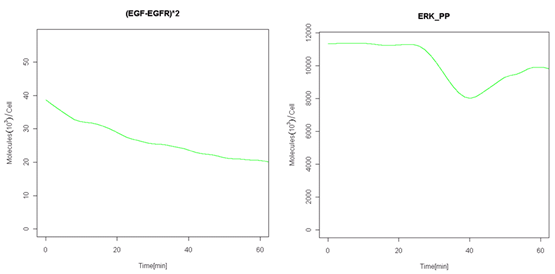

Supplement: Additional file 19 — Molecular profile of ERK-PP in response to signal from the EGF-EGFR complex as simulated by the abstract pi-calculus model, but without abstraction of the Grb module. [file 1752-0509-3-118-S19.TIFF]
