# Supplementary material for: Computational modeling of the EGFR network elucidates control mechanisms regulating signal dynamics
Source: BMC Syst Biol. 2009 Dec 22;3:118. doi: 10.1186/1752-0509-3-118 (PMC2807436; doi:10.1186/1752-0509-3-118)
Supplement: Additional file 3 — Behaviour of ERK-PP in response to complete inhibition of key reactions in the Grb2 module. [file 1752-0509-3-118-S3.PDF]

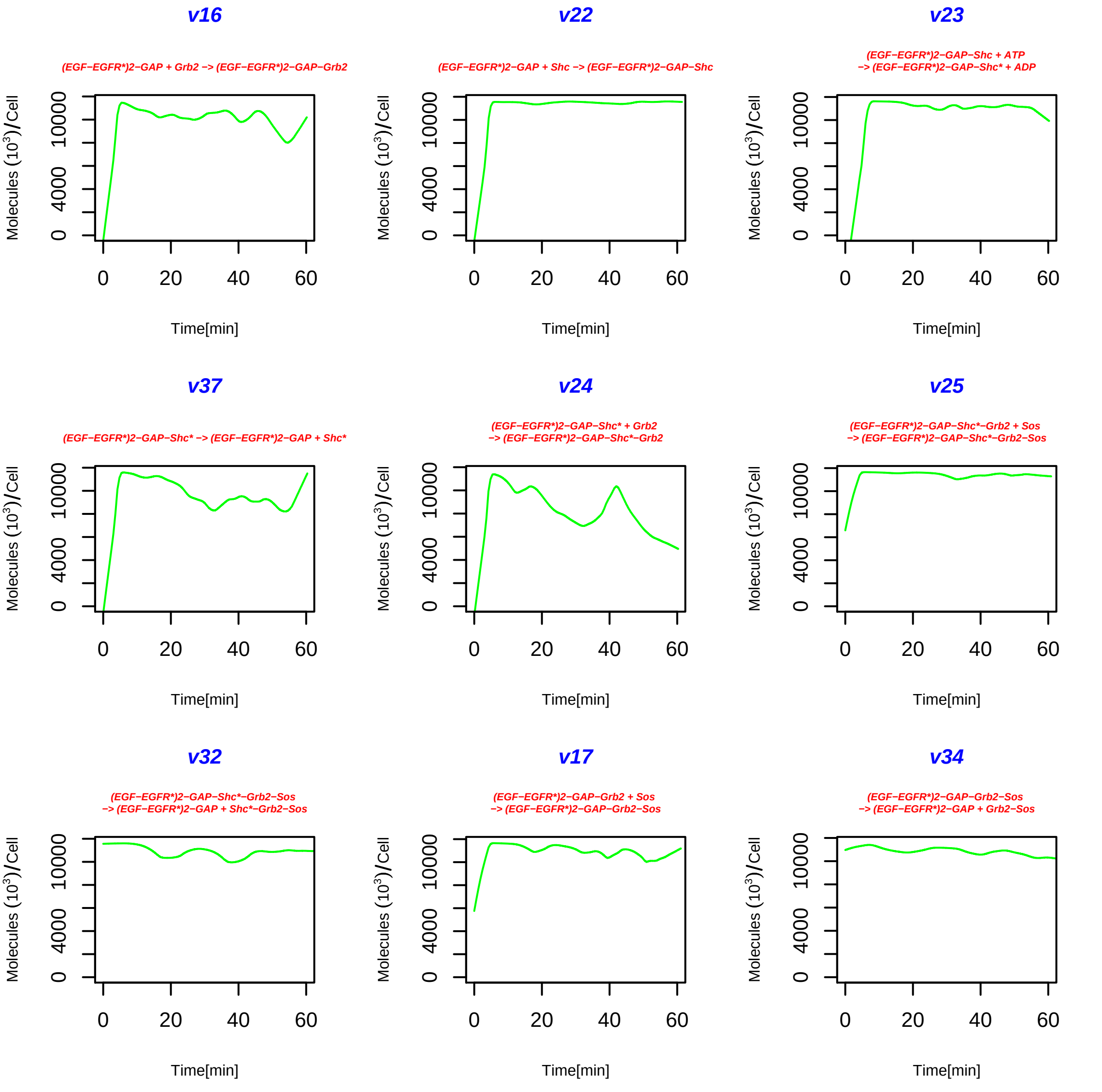
v16
(EGF−EGFR*)2−GAP + Grb2 −> (EGF−EGFR*)2−GAP−Grb2
v22
(EGF−EGFR*)2−GAP + Shc −> (EGF−EGFR*)2−GAP−Shc
v23
(EGF−EGFR*)2−GAP−Shc + ATP
−> (EGF−EGFR*)2−GAP−Shc* + ADP
v37
(EGF−EGFR*)2−GAP−Shc* −> (EGF−EGFR*)2−GAP + Shc*
v24
(EGF−EGFR*)2−GAP−Shc* + Grb2
−> (EGF−EGFR*)2−GAP−Shc*−Grb2
v25
(EGF−EGFR*)2−GAP−Shc*−Grb2 + Sos
−> (EGF−EGFR*)2−GAP−Shc*−Grb2−Sos
v32
(EGF−EGFR*)2−GAP−Shc*−Grb2−Sos
−> (EGF−EGFR*)2−GAP + Shc*−Grb2−Sos
v17
(EGF−EGFR*)2−GAP−Grb2 + Sos
−> (EGF−EGFR*)2−GAP−Grb2−Sos
v34
(EGF−EGFR*)2−GAP−Grb2−Sos
−> (EGF−EGFR*)2−GAP + Grb2−Sos
Molecules (10^3)/Cell
0
4000
10000
10000
0
20
40
60
Time[min]
