# Supplementary material for: Computational modeling of the EGFR network elucidates control mechanisms regulating signal dynamics
Source: BMC Syst Biol. 2009 Dec 22;3:118. doi: 10.1186/1752-0509-3-118 (PMC2807436; doi:10.1186/1752-0509-3-118)
Supplement: Additional file 7 — Behaviour of ERK-PP in response to complete inhibition of key reactions in the MEK module. [file 1752-0509-3-118-S7.PDF]

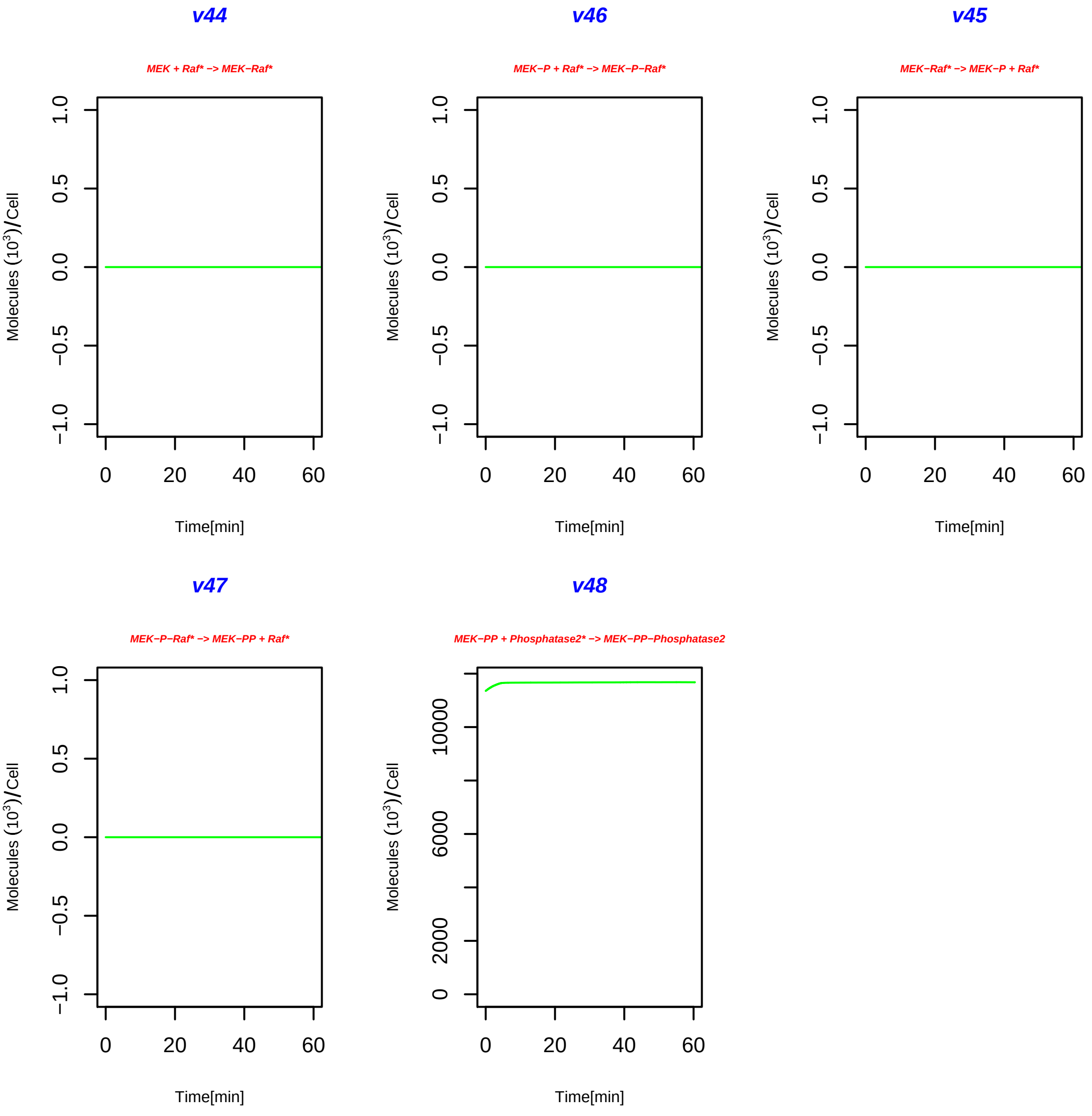
v44
MEK + Raf* −> MEK−Raf*
v46
MEK−P + Raf* −> MEK−P−Raf*
v45
MEK−Raf* −> MEK−P + Raf*
v47
MEK−P−Raf* −> MEK−PP + Raf*
v48
MEK−PP + Phosphatase2* −> MEK−PP−Phosphatase2
Molecules (10^3)/Cell
Time[min]
−1.0
−0.5
0.0
0.5
1.0
0
2000
6000
10000
0
20
40
60
